# Exploring potential risk pathways with high risk groups for urban Rift Valley fever virus introduction, transmission, and persistence in two urban centers of Kenya

**DOI:** 10.1371/journal.pntd.0010460

**Published:** 2023-01-12

**Authors:** Keli Nicole Gerken, Justinah Maluni, Francis Maluki Mutuku, Bryson Alberto Ndenga, Luti Mwashee, Caroline Ichura, Karren Shaita, Makena Mwaniki, Stella Orwa, Krish Seetah, A. Desiree LaBeaud

**Affiliations:** 1 Stanford University Division of Infectious Diseases Department of Pediatrics, Stanford California, United States of America; 2 Kenya Medical Research Institute Centre for Global Health Research, Kisumu, Kenya; 3 Technical University of Mombasa Department of Environment and Health Sciences, Mombasa, Kenya; 4 Stanford University Department of Anthropology, Stanford California, United States of America; Oregon State University College of Veterinary Medicine, UNITED STATES

## Abstract

Rift Valley fever virus (RVFV) is a zoonotic arbovirus that has profound impact on domestic ruminants and can also be transmitted to humans via infected animal secretions. Urban areas in endemic regions across Africa have susceptible animal and human hosts, dense vector distributions, and source livestock (often from high risk locations to meet the demand for animal protein). Yet, there has never been a documented urban outbreak of RVF. To understand the likely risk of RVFV introduction to urban communities from their perspective and guide future initiatives, we conducted focus group discussions with slaughterhouse workers, slaughterhouse animal product traders, and livestock owners in Kisumu City and Ukunda Town in Kenya. For added perspective and data triangulation, in-depth interviews were conducted one-on-one with meat inspector veterinarians from selected slaughterhouses. A theoretical framework relevant to introduction, transmission, and potential persistence of RVF in urban areas is presented here. Urban livestock were primarily mentioned as business opportunities, but also had personal sentiment. In addition to slaughtering risks, perceived risk factors included consumption of fresh milk. High risk groups’ knowledge and experience with RVFV and other zoonotic diseases impacted their consideration of personal risk, with consensus towards lower risk in the urban setting compared to rural areas as determination of health risk was said to primarily rely on hygiene practices rather than the slaughtering process. Groups relied heavily on veterinarians to confirm animal health and meat safety, yet veterinarians reported difficulty in accessing RVFV diagnostics. We also identified vulnerable public health regulations including corruption in meat certification outside of the slaughterhouse system, and blood collected during slaughter being used for food and medicine, which could provide a means for direct RVFV community transmission. These factors, when compounded by diverse urban vector breeding habitats and dense human and animal populations, could create suitable conditions for RVFV to arrive an urban center via a viremic imported animal, transmit to locally owned animals and humans, and potentially adapt to secondary vectors and persist in the urban setting. This explorative qualitative study proposes risk pathways and provides initial insight towards determining how urban areas could adapt control measures and plan future initiatives to better understand urban RVF potential.

## Author summary

Rift Valley fever is an important mosquito-borne zoonotic virus that can also be transmitted directly to humans via infected livestock. There has never been an urban outbreak of RVF, however, other arboviruses have caused devastating urban outbreaks driven by urban transmission cycles in vectors. This qualitative study aimed to explore and understand the potential risk of Rift Valley fever in the urban context from the perspective of high risk groups. RVF has a complex epidemiology and urban centers of endemic regions may be particularly vulnerable as they import large quantities of livestock for slaughter and milk to meet the high urban demand for animal sourced foods and have dense host and vector populations. This qualitative study provides insights from the opinions and lived experiences of urban high risk groups including slaughterhouse affiliates, livestock owners, and veterinarians. We also demonstrate risks specific to RVFV transmission and regulatory vulnerabilities that would impact the ability to detect RVF disease introduction. The results of this study are intended to guide future initiatives aiming to investigate urban potential of RVF and infer how urban disease ecology could differ from what has been previously demonstrated in rural areas.

## Introduction

Rift Valley fever virus (RVFV) is a zoonotic arbovirus with a transmission pathway reliant on suitable conditions for vectors overlapping with the presence of susceptible ruminant hosts. These processes are often driven by human activities including socio-cultural interactions with livestock and moving livestock in response to the demand for animal meat [1,2]. Communities that live in close contact with their livestock are more likely to experience the impact of large-scale outbreaks, and this has led to the assumption that risk is minimal for non-livestock associated communities. However, this has not been proven as RVFV can be transmitted by a wide range of arthropod vectors and opportunity for direct transmission independent of personal livestock ownership has been suggested [3–6]. Other arboviruses have caused devastating outbreaks in urban areas in response to altered climatic conditions and vector preferences, but there has never been a documented urban outbreak of RVF [7–10]. Limited insight on the unique and diverse challenges faced by urban areas may have resulted in these areas being omitted from mitigation efforts, field studies, and scenario planning [11]. As the continent of Africa, where RVF is endemic, is rapidly urbanizing and will soon lead the world in urban growth, a major proportion of future risk remains unquantified. [12].

RVFV seeding in new areas primarily relies on movement of infected livestock towards suitable vectors and susceptible hosts [13]. As demonstrated in the most dramatic transboundary spread to date from the African continent onto the Arabian Peninsula in 2000, large scale livestock movement from an endemic area to a naïve area can translocate RVFV and new transmission dynamics can be established [14]. Urban areas may be particularly vulnerable to RVFV as they have a high rate of livestock influx to meet the increased demand for animal-sourced foods (ASFs), must source from a wide geographical range, and have diverse aquatic habitats that could support breeding of competent RVFV vectors [15]. Our research group carried out a recent study in two urban areas in Kenya, Kisumu (Western region) and Ukunda (South Coast), and revealed a 2% human RVF seroprevalence with risk factors including consumption of fresh milk, along with vector exposure, in Kisumu as a potential source of exposure independent of livestock ownership [6]. Indeed, the high demand for ASFs in urban areas is well documented, and urban areas often have large tertiary markets where animals are imported, alongside a thriving practice in raising livestock within urban contexts [16]. Thus, there are two key populations of livestock in urban centers of RVF endemic countries: residential and regionally imported livestock entering the meat value chain. Furthermore, and relevant to RVFV transmission, processes of urbanization have occurred rapidly and often over a single generation. Cultural practices may shift risk priorities in the urban setting, for example considering, consumption of raw meat that was slaughtered in a high-volume facility compared to home slaughter [17]. Overall, more laboratory and field studies are required to understand and quantify RVFV risk via direct and vector transmission pathways in this setting.

Across all regions of Africa, where RVFV is endemic, recent urbanization over the past 60 years is unprecedented [18]. Urban populations are projected to more than triple over the next four decades and correspond to 21% of the world’s total urban population [19]. As people migrate to the city for more diverse employment opportunities, better access to healthcare services, and education opportunities, marginalized groups, and the poorest poor face extreme challenges. Inequalities in basic infrastructure result in a lack of piped water, inadequate drainage of sewers and surface water, and higher flooding potential [20], which may attract floodwater breeding *Aedes spp* that have been previously recognized as primary RVFV vectors important for outbreak initiation and could contribute to viral maintenance between outbreaks. However, field data for this phenomenon are lacking and experimental studies with floodwater *Aedes* demonstrate a major salivary gland barrier and successful transmission of only 14%, highlighting the critical role for other secondary vectors in the viral amplification phase of natural outbreaks [21,22]. RVFV has been detected in a wide variety of arthropod hosts and dominant vector species varies spatially and temporally, indicating the profound ecologic adaptability and high transboundary potential of this important pathogen [23,24]. Other arboviruses have proven to establish sustained urban transmission cycle as their primary vectors, *Aedes aegypti* and *albopictus*, now prefer breeding in urban areas despite dengue virus originating in forest sylvatic cycles [8,25,26]. Indeed, mosquitos can shift behavior and preferences in response to anthropogenic changes, [27] which was recently demonstrated by *Culex pipiens* (secondary vector) establishing a niche in the underground metro system of central London [28]. Overall, urban viral amplification is indeed plausible given the availability of hosts and wide range of competent vectors demonstrated in controlled and field conditions including other *Culex spp*., *Mansonia spp*., *and Aedes spp* [21,22].

In addition to understanding how changing ecological conditions alter risk, the social context of disease is equally important for RVF and other zoonoses. Public health messages that understand and incorporate high risk populations’ concerns, reality, and perceptions innately have higher success rates for reducing the burden of disease. As shown by the health messaging during the Covid-19 pandemic, urban populations may be willing yet simply unable to follow health messages when their livelihoods are also at risk [29]. For zoonotic livestock diseases, this effect is compounded for those that rely on livestock in urban settings and suddenly lose employment, market access, and purchasing power from closure of livestock markets and restriction of animal movement [30]. As RVF outbreaks have occurred in a variety of ecosystems [31], understanding the key difference between rural and urban risk could allow for inclusive, targeted, public health responses. Similarly, understanding risk of high risk groups perspective can help identify key research gaps for RVF human risk assessment. To understand the potential for urban RVFV introduction, transmission, and persistence in greater detail and to guide approaches that aim to develop understanding of urban RVF epidemiology, we carried out an exploratory qualitative study with high risk urban populations. The data from this study are intended to inform future urban field epidemiology studies, highlight regulatory vulnerabilities for transmission, and identify opportunities for early urban detection when regional risk is high.

## Materials and methods

### Ethics statement

This study in its entirety was approved by the Institutional Review Board (IRB) at Stanford University (IRB-57869) and the Technical University of Mombasa IRB (TUM ERC EXT/004/2019 (R)).

### Study design

**Fig 1:**
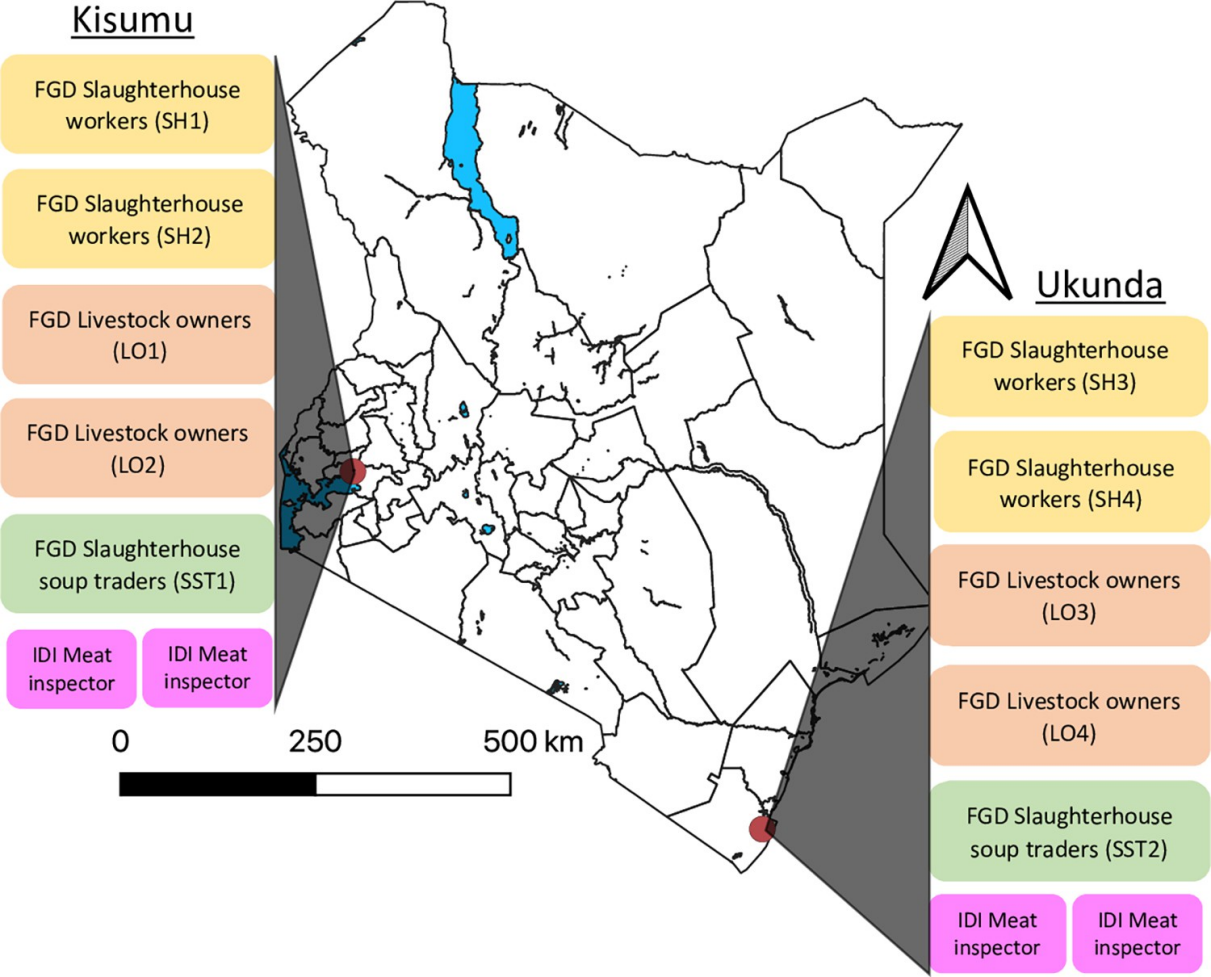
Study design at urban sites Kisumu and Ukunda, Kenya. Acronyms: FGD: focus group discussion, IDI: In-depth interview, SH: Slaughterhouse, LO: Livestock owner, SST: Slaughterhouse soup trader Link to base map: https://africaopendata.org/dataset/kenya-counties-shapefile.

We carried out an explorative qualitative study across two urban study sites in Kenya in August and September 2021 (Fig 1). Ten focus group discussions (FGDs) were conducted to understand the consensus of groups that we considered to be at highest risk for RVF in urban settings: slaughterhouse affiliates and livestock owners. To triangulate data from each respective slaughterhouse, veterinarians who were meat inspectors responded to in-depth interviews (IDIs) on their perception of urban RVFV risk, experience containing zoonoses such as RVF, and predictions for future urban outbreak responses.

### Study objectives

This study’s main objective was to explore potential pathways of RVFV entering and transmitting within urban centers. Specifically, we aimed to understand how high risk groups that were associated with livestock compared their personal risk in the urban setting to rural areas, the preventive measures already in place at our two urban sites, and how current knowledge and experience with RVFV and other zoonotic diseases may impact implementation of future disease countermeasures. Question guides were created to ensure full coverage of the study objectives and homogeneity between the study sites. The guides were piloted with smaller groups (3-5 participants) from adjacent towns to gather baseline information and inform probing techniques for the moderator to cover. We considered the priority livestock species for RVF risk to be cattle, sheep, goats. We also included questions regarding animal products that could potential carry RVFV, including fresh milk.

### Study areas

We purposefully selected two urban study sites, Kisumu, and Ukunda, to carry out this qualitative study as they were established as part of the parent study this project was associated with and supported by. Inclusion of both sites allowed us to gain insight from two geographically distinct and culturally diverse urban centers in Kenya. Kisumu city is located on the shores of Lake Victoria in Western Kenya and Ukunda town is an emerging urban center located on the South coast of Kenya. At both urban sites, nearly all animals are slaughtered in designated slaughterhouses as both of these areas are managed as a municipality according to the 2012 Urban Areas and Cities Act of Kenya [32]. Ukunda represents an emerging urban center and population growth has skyrocketed since the early 1970s in response to tourism. The Kisumu site represents a larger, well-established urban city. Livestock ownership at both study sites has recently been documented to be overall low (9% poultry, 6% dairy cows, 1.4% dogs, 3% cats, 1% goats, 0.4% beef cattle, 0.2% sheep, and no pigs) and, this urban community had a 2% lifetime RVFV exposure prevalence. In this study, risk factors were linked to lack of piped water in the home, consumption of raw milk in Kisumu, and seeing goats around the home in Ukunda, though these risks were not independent of vector exposure, which was near ubiquitous [6].

Kisumu (0°5’ 15.2247800 S, 34°46’ 22.328400 E) is the third largest city in Kenya and a business hub at an altitude of 1,100 meters above sea level, and had a population of 610,082 inhabitants in the 2019 census [33]. Temperatures are on average 23°C (16-33°C) and total rainfall averages 1,966 mm per year with a relative humidity of 63% [34]. Western Kenya was previously classified as low risk for RVFV outbreaks; however, during the 2018 outbreak, RVFV was detected in adjacent Siaya County of the Western region for the first time [35,36]. Kisumu city has a total land area of 2,085 square kilometers and has a per square kilometer density of 544 people. However, our livestock owner study participants were only recruited from Kisumu East sub-county which has a total land area of 142 square kilometers and a per square kilometer density of 1,560 [37].

Ukunda (4°16’ 38.8992” S, 39°34’ 9.0012” E) is located on the South coast of Kenya 30 km South of Mombasa at an elevation of 24 meters above sea level with an estimated population of about 100,000 people [20]. Ukunda is the largest urban center in Kwale County and is an area of more recent urbanization in Kenya and has temperatures on average 28°C (23-34°C) with total rainfall averaging 1,060 mm per year and a relative humidity of 74% [38]. Kwale County has been involved in at least 10 national RVFV outbreaks [39] and recent studies classified Kwale County among the highest level risk areas for epizootic RVFV transmission in Kenya [39]. Ukunda town covers a land area of 25.5 square kilometers and has a per square kilometer density of 2,060 people [37].

### Participants and participant recruitment

We worked with key informants and community health volunteers to identify a representative sample of the urban livestock community within our study boundaries. We aimed to recruit seven to ten participants for each FGDs aggregated by type of livestock association (slaughterhouse workers, slaughterhouse soup traders (described below), and livestock owners). Participants were identified two weeks prior to being invited to participate in the FGDs and attempts were made to capture an equal number of men and women of all age groups and religions.

### Slaughterhouse workers

Four groups of slaughterhouse workers were recruited from the two main ruminant slaughterhouses at each site, and we invited participants that carried out all slaughterhouse activities including the slaughterman, skinners, and meat filleters. In Kisumu, participants were recruited from Mamboleo and Rabuor slaughterhouses and in Ukunda, participants were recruited from the Mwabungo and Bongwe slaughterhouses. The managers were invited to be a part of the discussion if they participated in the daily activities on the slaughterhouse floor. To be included in this group slaughterhouse employees must have worked at the slaughterhouse for a minimum of six months. We also confirmed that the two main slaughterhouses at each site primarily served the urban meat market, and therefore were likely to have a high demand and volume of slaughtered animals each day.

### Slaughterhouse Soup traders

We identified individuals who purchased various byproducts daily from each of the participating slaughterhouses, including the heads, hooves, offal, and blood and invited two groups total, one from each site, to participate in this study. These individuals handle the animal products shortly after slaughter and the most common practice of this group is to prepare them into soup to sell by boiling the heads and hooves, so we collectively refer this group as the “slaughterhouse soup traders” (SSTs) throughout this manuscript. Certain members in this group also purchase blood to either make local blood sausage (mtura) or resell privately to farmers as livestock feed. To be included, SSTs must have been a consistent customer for either blood or byproducts from the slaughterhouse. Only two groups total, one from each site, were selected as there was a limited number of eligible individuals that carried out these practices at the selected slaughterhouses.

### Livestock owners

Four groups of livestock owners, two at each site, were recruited for this study with the help of local elders that served as key informants. Ownership in this study was defined as possessing at least one ruminant (cow, sheep, or goat) and we specifically aimed to identify participants involved in local dairy production and business. Although livestock owners with dairy animals were targeted, participants who owned multiple small stock (sheep and goats) for meat production were also invited. All cattle owners collected milk from their female animals. At each of the study sites, participants were recruited that resided within the urban area and therefore shared similar urban farming practices. In Kisumu, only livestock owners that primarily lived within Kisumu East sub-county were considered for inclusion and these individuals primarily lived within the Migosi estate neighborhood. In Ukunda, livestock owners were only recruited from homesteads near (within three kilometers) to the city center.

### Data collection

The slaughterhouse FGDs took place in a private room or designated area at the respective slaughterhouse. Livestock owners were invited to meet at a central public location that was identified by key informants and deemed an acceptable travel distance from the participants’ homes. At enrolment, basic demographics were collected for each study participant. FGDs were carried out in Kiswahili except for the SSTs group in Kisumu, which was conducted in the local language, Dhuluo, which was preferred by all participants and spoken natively by the moderator. All groups and interviews were audio recorded with a Sony IC audio recorder (ICD-PX470) and transcribed verbatim in English by the moderators and their assistants within one week of each FGD or interview being carried out so that transcripts could be reviewed as field work was ongoing and inform assessment of data saturation. Randomized quality control spot checks (identified by line number random number generator) were completed for each transcript by a second transcriber and any discrepancies in translation were discussed with the full team.

The first author of this study was present at all FGDs serving as the note taker and personally led the IDIs in English. Confirmation of cultural acceptance of this dynamic was independently determined by the FDG moderators, our key informants, and the Kenyan primary investigators (Mutuku and Ndenga) at each site. Decisions for leading the IDIs in English were centered on the interviewer sharing a common professional background (veterinarian) with the IDI participants, that they were fluent in English, and had an established rapport from previous interactions with this researcher. Positionality was actively reflected on throughout development and execution of this study by all authors.

A moderator and assistant moderator were trained from each site and the moderator from the first site (coast) was physically present for the piloting and training at the second site (West) to ensure consistency in data collection. The moderators from each site were residing in the same area and belonged to the same tribe as most of the FGD participants. Team training was carried out over two weeks and focused on understanding how the study objectives related to RVFV epidemiology and highlighted the importance of minimizing responder bias.

### Data analysis

Transcripts were read from which a codebook was developed for thematic analysis. The transcripts were uploaded into NVivo (*Version 1*.*5*, *4577*) and transcripts were coded by two independent coders according to the codebook and organization of codes was discussed in real-time. The coded transcripts were verified by a mutual third coder to ensure consistency.

All FGD data were analyzed together for a collective understanding of urban perspectives and where appropriate, differences between the groups and two study sites were noted. A thematic analysis was carried out using open coding, axial coding, and selective coding to identify prominent themes in the qualitative data relevant to the study objectives. IDIs provided data triangulation from a different perspective at the slaughterhouse and IDI analysis focused on themes identified in the FGDs. Text has been summarized in the results section from detailed ethnographic accounts.

### Ethics

This study in its entirety was approved by the Institutional Review Board (IRB) at Stanford University (IRB-57869) and the Technical University of Mombasa IRB (TUM ERC EXT/004/2019R). Field activities were initiated after receiving a research permit from the Kenyan National Commission for Science, Technology, and Innovation (NACOSTI). Meetings with local administrators (County Commissioners, Deputy County Commissioners, Assistant County Commissioners, Chiefs and Assistant Chiefs) were held to obtain their approvals. Meetings with the community were not conducted due to the then existing Ministry of Health COVID-19 guidelines. Participants were sensitized during recruitment and invited to participate on a voluntary basis. All participants received the same briefing during consenting and were allowed to ask any questions to the group or privately to the moderator. Before beginning, participants were informed that the study was focused on Rift Valley fever and their responses could be used to inform policy and disease control in their area. To decrease the risk of responder bias, questions specific to RVF disease dynamics were deferred to the end of the FGD. The assistant moderator spent approximately ten minutes explaining the study procedures and informing participants their rights as a research study participant. Understanding of informed consent was assessed through a checklist at the end of the consent form and each participant individually signed a consent form and was given a copy. All participants were above Kenya’s adult consenting age of 18 years and were compensated for their lost time as an opportunity cost associated with their business and transport fees to travel to the designated meeting point.

## Results

Ninety-one participants from ten different FGDs (n = 87) and four IDIs (n = 4) were enrolled in this qualitative study and participated until the end of their respective sessions. A summary of FGD participant demographics is represented in Table 1. Data saturation was achieved in the final FGDs from the slaughterhouse groups and livestock owners, and this was confirmed by codebook developers, the primary investigators, and moderators as no additional themes were added after the final groups that occurred in Kisumu. Indeed, despite geographical separation, there was significant similarity between group dynamics and responses across the two study sites, and any major differences have been noted below under each theme in the thematic analysis. FGD length varied from 46 to 94 minutes. At the coast site (Ukunda), it was challenging to identify female livestock owners despite the best efforts from key informants and use of the snowball sampling technique. Given the gender ratio for the recruited livestock owners of 13 male to four female participants in Ukunda, we decided to organize one group of only men and another group with 50% men and 50% women. All slaughterhouse workers at both sites were men; however, women were involved at the slaughterhouse as SSTs. All SSTs in Kisumu were women and the years of experience in this group was comparable to other slaughterhouse staff. The IDIs were with two men and two women and to protect the identity of these meat inspectors, age and location has not been reported.

**Table 1:** Focus group discussion participant demographics.

	Ukunda (coast)					Kisumu (West)					
Demographic	SH1	SH2	SST1	LO1	LO2	SH3	SH4	SST2	LO3	LO4	Total
n = (Gender M/F)	8 (M)	9 (M)	4 (M) 6 (F)	8 (M)	5 (M) 4 (F)	9 (M)	8 (M)	8 (F)	5 (M) 5(F)	4 (M) 4 (F)	60 (M) 27 (F)
Median age in years [range]	38 [32-50]	37 [19-66]	37 [30-62]	51 [26-75]	48 [20-78]	35 [24–49]	34 [26-56]	52 [22-70]	46 [28-69]	52 [22-70]	41 [19-78]
Median years of experience [range]	2 [10–13]	14 [1–30]	6 [1–10]	11 [2–28]	10 [4–45]	15 [1–30]	6 [3–10]	9 [5–40]	14 [2-52]	9 [5–40]	
LO: Number that owned at least 1 dairy cow (%)				7/8 (88%)	6/9 (67%)				5/10 (50%)	6/8 (75%)	
SH: Primary roles of participants (n =) SSTs: Primary animal product purchases (n =)	Manger (2) Skinning (6) Clean intestines (2) Slaughterman (2) Cleaning (2) Filleter (2) Source cattle (1)		heads, hooves, tails, blood (n = 1)			Manager (1) * Skinning (9) Slaughtering (2) Intestines (2) Cleaner (2) *Also skinning		heads, hooves, tails, blood (n = 1)			

SH: slaughterhouse; LO: livestock owners; SST: slaughterhouse soup traders

M/F: male/female

### Influences of urban RVFV risk pathways for introduction, transmission, and persistence

**Fig 2:**
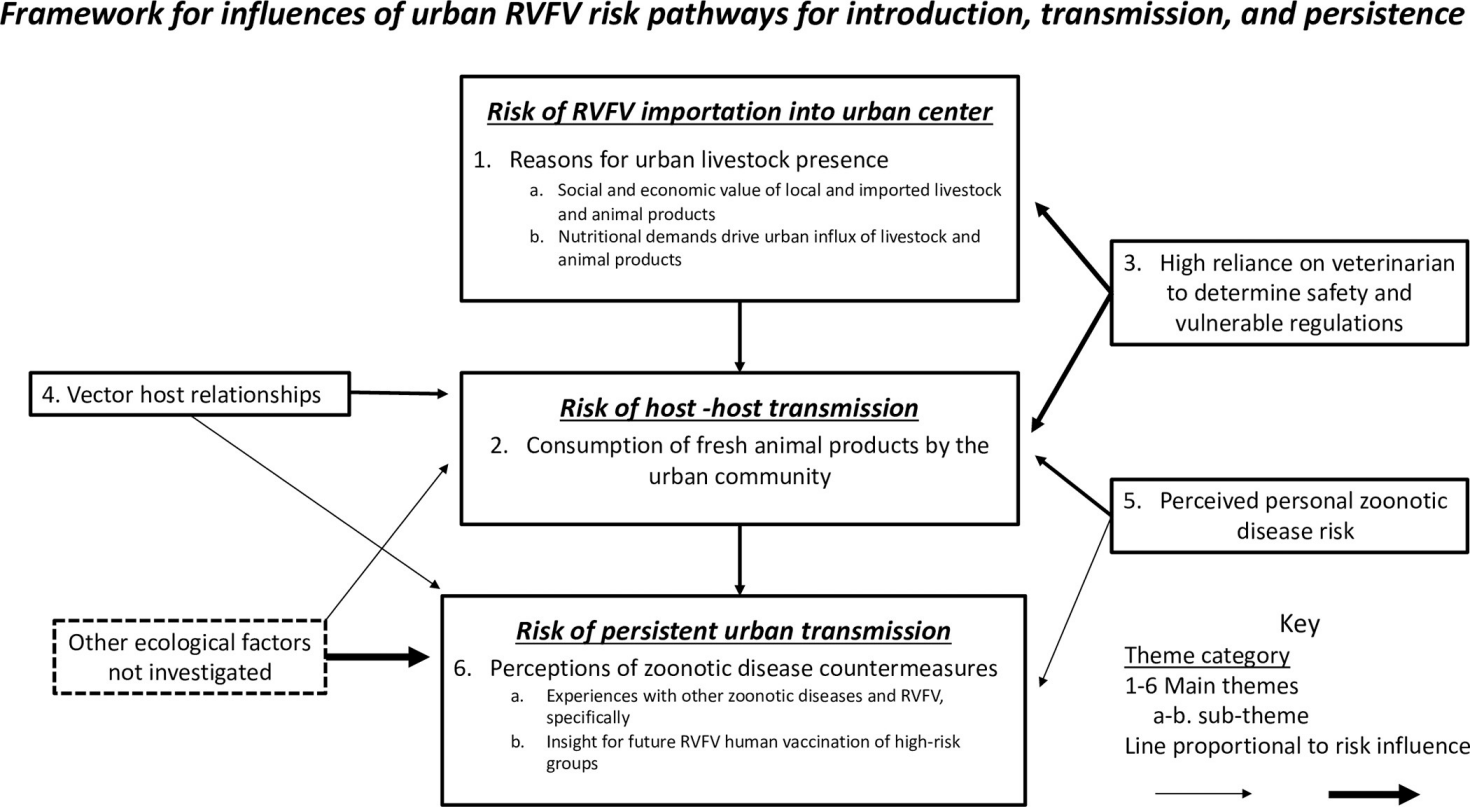
Theoretical framework for influences of urban RVFV risk pathways for introduction, transmission, and persistence.

Qualitative data analysis was focused on themes that were relevant to the study objectives and six prominent themes [1–6] emerged from the qualitative data. Data has been organized according to the theoretical framework presented in Fig 2, in which each theme influencing risk classified under three risk categories: introduction, transmission, and persistence. Key messages for each theme have been summarized and notable quotations direct from the participants are organized under each theme.

### Risk of RVFV importation into urban center

1. **Reasons for urban livestock presence**
  a. **Social and economic value of local and imported livestock and animal products**

In the urban setting, both locally owned and imported livestock and their products were referred to as a business opportunity, but they still retained personal sentiment. At the household level, selling animal products paid children’s’ school fees and other daily expenses, while selling livestock was reserved for financial hardships or for marriage payment. At the slaughterhouse, workers expressed pride in providing safe protein for their comunities and at least five women in one of the Kisumu livestock owners’ groups and all in the SSTs group regarded that they were proud of their ability to independently generate income. The SSTs demonstrated that all animal parts can be used for income generation including the heads, hooves, and blood, and both groups noted the importance of being able to provide customers with a lower cost protein option. Aside from human consumption, blood that was less fresh could be sold to farmers as feed for pigs (Kisumu SSTs) and poultry (all SSTs). All slaughterhouse sales were conducted as private business and the Ukunda slaughterhouse groups highlighted the businessperson who purchased the live animal at market would lose significant investment if a full carcass was condemned by the inspector and it was the businessperson who dictated the day of slaughter for each animal. We observed the holding pen for cattle and small stock at one of the slaughterhouses and the pen for cattle was approximately 5x5 meters holding approximately 40 animals and therefore, we considered these conditions to be dense and high volume, but the slaughterhouse groups did not state this when asked the details of the holding pen. In addition to the urban slaughterhouse providing a central location for business, an additional social benefit was significantly reduced local livestock theft given the requirement for an official stamp on all marketed meat

“*These slaughterhouses have helped in so many ways*, *cattle theft is very low because when you steal an animal after slaughtering*, *it isn’t stamped*. *You’ll have to explain where you got the animal*, *so even the thieves fear*, *and it helps the livestock owners too” – Ukunda SH2 R5*

**b. Nutritional demands drive urban influx of livestock and animal products**

The major drivers for urban livestock influx were related to urban nutritional demands of humans and animals. We confirmed livestock and animal products must be sourced from distant locations to meet the high urban demand for ASFs and imported live animals had numerous opportunities to mix with urban resident animals.

Arrivals at the slaughterhouse were not slaughtered immediately on arrival and instead were kept in holding pens with holding times dependent on orders received from local butcheries. Both Ukunda and Kisumu slaughterhouse workers noted that animals in the holding area never “ran out” as they were always replaced with new arrivals. In Ukunda, animals primarily walked overnight to arrive at to the slaughterhouse, even if their origin was more than 200 kilometers away and waited in holding pens up to one month. In Kisumu, animals waited “at most two weeks.” While in holding, animals reportedly grazed and drank water around the slaughterhouse grounds as they recovered from their transport.

The livestock owners group provided insight on other commodities, such as milk, that entered the urban centers in large quantities in response to the unmet local demand. In Kisumu, milk was noted to specifically arrive from Eldoret and Nandi (cities in Rift Valley Province). In Ukunda, milk came from nearby Shimba Hills (rural town in Kwale County) and Lunga Lunga (border town with Tanzania). This milk was reported to quickly sell out as the demand for fresh milk from urban consumers was very high. Livestock owners were aware of high competition in milk business but did not regard it as a disruption to their local sales. They agreed they could not keep up with the demand for fresh milk alone.

In addition to human nutritional demands driving influx of livestock and animal products for consumption, our livestock owner participants reported that at certain times of the year livestock from other places would arrive near to the urban sites to graze from far away when there was drought at their home. These situational grazers were present at both study sites, but were more common on the coast of Kenya, near to Ukunda, when large herds were reported to arrive when they did not have grass available at their respective homes in Kajiado and Garissa counties (two RVFV hotspots in Kenya). Each time this practice was mentioned in the livestock owner FGDS, at least one other individual noted the importance of keeping their animals separated from these visiting animals.

“*They stay here until rains start*, *and they call back home and ask if the rains are there or not*. *If they are told rains are there*, *they go back but when they are told the situation is the same*, *they stay here*.*”*

– *Ukunda LO2 R4*

“*I think before December we will receive those visitors*. *For example*, *I watched the news yesterday*, *and I found out that in Northeastern it is dry to an extent*, *and that the cows are dying*, *so when there is no food*, *they start coming down here*.*”*

– *Ukunda LO2 R6*

Meanwhile, urban livestock were reported to have limited grazing areas and/or affordable zero-grazing feed options which meant they either had to be grazed in the peri-urban area or to scavenge locally on urban market vegetable waste. Peri-urban grazing provided an opportunity for mixing (and sharing vectors) with imported livestock and market scavenging carried other health risks. A livestock owner in Kisumu stated that when her cattle encountered animals she didn’t recognize, she was careful to pick any new ticks off the animal post-grazing. Approximately half of all livestock owners at both sites reported to release their animals daily near to marketplaces to scavenge on vegetable scraps as this was deemed necessary for animals to meet their daily nutritional needs. There were additional challenges associated with roaming urban livestock including being detained by police, poisoning, neighbors abusing animals with knives, dog attacks, snake bites, and consumption of plastic bags. Consensus was that all participants owning livestock had difficulty finding enough feed for their animals.

“*So*, *you know when they bring the cattle here*, *we don’t know the problems they have*, *so when the animals go to graze out there*, *they mix with the local animals*. *So*, *when they mix there if they have diseases it is transmitted to the others*.*”*

– *Kisumu SH3 R1*

“*You can see our cows feeding*, *but they are not strong*. *The strength we give our cows is by releasing them to struggle through the road to eat people’s kales along the road*, *and to eat garbage that has been pushed together so that they get satisfied*.*”*

– *Ukunda LO1 R3*

### Risk of host -host transmission

2. **Consumption of fresh animal products by the urban community**

Fresh animal products, including milk and fresh blood (Kisumu), were only sometimes viewed as risky and were reported to be important for human health. Local fresh milk was preferred, particularly for children and babies, because of concerns for chemicals in commercially packed milk. Trust in fresh foreign milk that arrived from surrounding regions was lower than local milk, as it was believed that foreign milk had chemicals, adulterants, and water as this was probably the only way for them to compete with commercial milk prices.

Among the dairy owning livestock owners, it was understood that boiling milk was important for health, but local fresh milk was still sold un-boiled, and it was to be the consumer’s decision to boil the milk or not. At least two other livestock owner participants agreed with this statement and added that some people drink milk direct from the cow and they do not have any health consequences, so therefore, “the dangers are higher than the truth.” However, most participants in each livestock owners’ group recognized that un-boiled milk could cause disease and a commonly known consequence of raw milk consumption was diarrhea. Fresh milk was not just for food but also used as medicine, and human tuberculosis (TB), burns, HIV, and arthritis were specifically mentioned to be treated with fresh milk.

The slaughterhouse workers shared that blood was collected in plastic jugs and sold to the SSTs who all confirmed that these products were purchased. The meat inspector veterinarians from these two slaughterhouses in Kisumu affirmed the exchange and recognized that because the blood was pooled from multiple animals before dividing the carcasses, it was difficult to remove any if a contributing animal to the blood pool was condemned. Fresh, raw blood purchased from the slaughterhouse was also said to be consumed as a cure for health problems in Kisumu. The SSTs assisted community members to receive these products on request, but we did not confirm if the participant’s (SST R3) remark that it was doctors suggesting this practice was in reference to conventional doctors or traditional doctors. It was also unconfirmed if the meat inspector participant referencing this was in respect to doctors at official hospitals or traditional places of healing, as we did not probe these participants to explain further.

“*You know blood from an animal is normally warm*, *then they drink it*, *the following day they come as well and might go even for a week and then he regains blood quickly that is what the doctor says*. *He is just someone who has been directed by a doctor that go and drink raw and direct milk from a cow*.*”*

– Kisumu SSTs R3

“*People believe that that blood boosts their immunity*. *When they go to the hospital*, *they are told*, *especially pregnant mothers*, *to take blood from the slaughterhouse*.*”*

– Vet 3

3. **High reliance on veterinarian to determine safety and vulnerable regulations**

Overall, preferences for slaughtering and decisions for meat consumption were highly focused on the physical presence of the meat inspector veterinarian at slaughterhouses. Trust in the veterinarian was said to overpower any personal opinions, and their “final word” was mentioned by all slaughterhouse groups when prompted to discuss food safety. All slaughterhouse groups repeatedly assured the moderator that all meat products are inspected, affirming that if the veterinarian had examined a product, then it must be safe for consumption.

The veterinarian’s post-mortem examination, rather than live animal examination, was deemed the best way to determine if a meat is safe. However, decisions for when to notify the veterinarian of sick animals at the slaughterhouse varied between and within groups and the animal was either slaughtered for post-mortem examination or the veterinarian was called immediately to examine the live animal. The SSTs who had no direct interaction with the meat inspector, also perceived their products (the carcass heads) to be free of disease if the veterinarian had visually examined them.

“*I don’t think there is any risk because the meat is inspected by a doctor*. *It is confirmed that its healthy*, *so I don’t feel at any risk”*

*- Ukunda SSTs R2*

Animals were sometimes treated for illness before slaughter and all four IDI respondents reported that every urban slaughtered animal should receive an antemortem examination the day before. They all also stated that they were required to respond to emergency slaughter situations to determine safety for slaughter if an animal arrived sick or injured. It was also mentioned in Ukunda that if the treatment was with antibiotics, a withdraw period of two weeks was observed while the animal waited at the slaughterhouse.

Despite the presence and active role of veterinarians in public health, regulations were reported by our participants to be prone to violations particularly around meat certification and slaughtering of sick animals. The slaughterhouse workers group firmly affirmed that it was illegal for any dead animals to enter the slaughterhouse premises. However, at least two participants in the livestock owners group anecdotally described that in recent years, these requirements have been circumvented when meat from sick or dead animals was mis-marketed and mixed in with an officially approved animal. One example was given by a slaughterhouse worker from Kisumu that shared a loophole for slaughtering dead animals was if the owner reported a traumatic accident (for example, being hit by a car). In such case, they preferred to slaughter the animal quickly to determine if any meat was salvageable. The livestock owners’ group in Kisumu, rather than the slaughterhouse group, also stated that there are individuals who slaughter sick animals outside of the official system and they may have access to fake or corrupt stamps of approval on their meat. One of the main drivers for corruption in meat certification was explained to be the huge financial loss to the owner of the animal.

In the case of an official veterinary meat inspector condemning part or all a carcass, this process had significant professional oversight. In all four IDIs, participants highlighted the importance of strict follow up when they condemn a whole or part of a carcass and each participant shared at least one personal story of a community members becoming sick or dying after eating meat that was purchased through a regulatory loophole and therefore, they were strict with follow-up. Notably, one of the meat inspectors from Kisumu revealed that blood is one potential product that can bypass the condemnation process because it is usually pooled early in the slaughter process with many other animals and distributed before the carcass inspection later occurs.

“*It might not even be the veterinary’s stamp*. *The butchery people also have their own rubber stamps*, *and you will find that after the veterinarian has stamped theirs on one part of the animal*, *they then stamp on the other pieces or parts and mix with the other pieces*.*”*

– Kisumu LO3 R8

“*There are those who imagine losing a whole cow*, *they then make a deal with the slaughter guys*. *They are then given something little*, *then the cow is taken to the slaughterhouse and slaughtered*.*”*

– *Kisumu LO3 R10*

Vulnerabilities were also present in animal movement restrictions. Data from an Ukunda IDI explained the process for requesting a movement permit and that during high risk times issuing of movement permits was suspended. Anyone that aimed to bring a live animal across County lines had to obtain a movement permit from a veterinary official. However, they recognized that movement within the County was still allowed and that everyone realized some animals still move at night undetected or use “short cuts.” In Kisumu, an IDI participant added that while there was an official inspection point at border control with Tanzania, people moved their animals through an unofficial corridor or traveled at night.

4. **Vector-host relationships**

Participants from four out of ten groups connected vectors as a means of infection transfer to humans and animals and explained abundance to be associated with host presence, weather, and availability of mosquito breeding sites. Flies and mosquitoes were said to be more numerous near to places livestock are kept and the wet conditions of enclosures were said to be responsible for this effect. Tsetse flies and tick abundance was also noted to be associated with the presence of animals near to homesteads. In Ukunda, one livestock owner participant recognized that if a tick bites another animal and then bites you or your animal, you could get the same disease. Additionally, livestock owners noted that when mosquitoes bite animals they (the animals) can also get malaria, and that their risk of disease transmission must be higher because they do not sleep under mosquito nets.

“*Local people can be bitten by mosquitoes; mosquitoes could have bitten an animal then it comes and bites you and leaves you with some diseases it got from somewhere*.*”*

– *Kisumu SH4 R5*

5. **Personal zoonotic disease perceived risk**

FGD participants were generally aware that diseases could be transmitted from animals to humans and stated the name of several zoonotic pathogens including anthrax (KSM LO4), bilharzia (KSM LO3), and tetanus (KSM SSTs). When groups were asked about their personal risk of zoonotic disease, they related their level of risk to common practices and compared risk amongst each other in the group and to rural areas.

The Ukunda SSTs knew they were at risk for zoonotic disease but said that “there is no business which has no risks,” and that even the fire for cooking the products was a health risk for them. The Kisumu SSTs recognized that the slaughterman could become infected with an animal disease by inhaling the “hot blood” from the animal, and they concluded that while they do have some contact with the blood and animal products, the slaughterhouse workers were touching the animals more, so they were at higher risk. When risk was compared among the slaughterhouse workers groups, both sites identified the slaughterman to be at the highest risk, but noted that indeed, “everyone touches the blood.”

The perceived occupational risk of slaughterhouse workers was heavily focused on hygiene which nearly all participants determined to be superior to rural areas because of urban running water availability. However, one participant in a Kisumu livestock owners group felt that urban populations could be at higher risk because of congestion and air pollution. For their protection while working, slaughterhouse workers most regarded their gumboots and a clean laboratory coat to be important in risk reduction, and this was required for reporting to work each day. At both sites, the older slaughterhouse workers (> 50 years), recalled when there were less regulations for slaughtering and believed that the new measures reduced health risk. Slaughterhouse workers also mentioned that their required quarterly occupational health checks, including deworming and typhoid vaccinations, were important for maintaining their personal health.

Slaughterhouse workers could recognize disease in live animals and explained that examining live animals was most important at point of purchase in marketplaces. The animal’s hair and eyes were examined, and other general signs of sickness were related to the animals’ ability to walk, drink water, and eat normally. Post-mortem, slaughterhouse workers could also sometimes presume when certain parts of the animal would be condemned even before the veterinarian arrived, but they had to wait for instruction and oversight. The slaughterhouse workers commonly recognized bruised meat, medication injection sites, parasites in organs and in the intestines, and abnormal livers, knowing these portions would be condemned. No groups at the slaughterhouse referred to a strong link between these reasons for carcass condemnation and their personal risk of zoonotic diseases.

“*You find the hairs have stood on end*. *It is difficult to know if it’s the liver that is bad or what*, *and it’s just there*, *and later when you slaughter it you find that the liver is bad*.*”*

– *Kisumu SH3 R1*

Overall, consensus was reached in all groups that they did not have enough knowledge regarding zoonotic diseases, and they requested more training and demonstrations because they feared these diseases. Groups specifically asked to understand transmission pathways, methods of prevention, and disease names so they could determine their risk when they heard about them. Slaughterhouse workers noted that they touch the blood with their bare hands, but the inspector used a hook, so therefore they have higher risk. Anthrax was specifically mentioned in Kisumu as one of their greatest fears and the slaughtermen knew not to slaughter if they saw abnormal bleeding in a live animal. One slaughterhouse worker in Kisumu suggested more collaboration between the meat inspector and the slaughterhouse workers to determine if it was safe to slaughter an animal. In triangulation, meat inspectors reported that they were dedicated to public health and the wellbeing of the slaughterhouse workers, but the slaughterhouse workers had low motivation to learn about the diseases and were focused on business.

“*I have an opinion*, *if it’s possible*, *people like us*, *make us learn so that we know all the diseases because we just keep cows and only the meat inspector knows the diseases”*

- Ukunda SH2 R6

“*My worry is that*, *during the process I don’t know what kind of diseases I am exposed to and when it’s a disease that can be transmitted in the blood*, *then we play with blood every day*.*”*

– *Ukunda SH1 R7*

### Risk of persistent urban transmission

6. **Perceptions towards zoonotic disease countermeasures**
  a. **Experiences with other zoonotic diseases and RVFV specifically**

At least one participant from all FGDs shared a personal experience with a zoonotic disease control measure, and this contributed to the groups’ self-assessment of risk. At the household level, three livestock owners shared their practices for reducing mosquitoes around their animals including draining stagnant water and burning branches to create smoke. Livestock owners also shared experiences with animal movement bans and reactive vaccination for RVFV.

A slaughterhouse in Kisumu also reported they had grazing restrictions for animals that entered from far away and sometimes kept these animals in a pen adjacent to the other animals. At least five slaughterhouse workers recalled animal movement restrictions “nearly closing the meat sector,” which greatly impacted their livelihoods. When we asked about other reasons for movement restrictions, three slaughterhouse groups recalled foot and mouth disease outbreaks to be the most common cause for movement restrictions. In general, movement restrictions were understood to stop disease introduction and workers were motivated to comply with regulations because they understood that if the disease arrived, slaughter bans would further impact their livelihoods.

For RVF specifically, at least one person in each of the slaughterhouse workers and livestock owners group recalled a prior RVF outbreak. In Ukunda, the livestock owners noted that during the last major outbreak, RVF was present in the county, but was not seen as an urban threat. One of the meat inspectors confirmed this and expressed their own personal frustration for the lack of One Health approaches at the start of last RVF outbreak, sharing their experience with cooperation from the human health sector begin low until staff at their local hospital personally detected the first human case in the County. However, this point was not expressed by the other FGD or veterinarian IDI participants. The majority of FGD participants who knew about RVFV understood it to be a serious disease for livestock and reported that RVFV health messaging focused on avoiding meat and milk consumption, particularly if these products were sourced from the Rift Valley region of Kenya. Most recently, during the 2018 outbreak in Siaya (Western Kenya), three participants in the Kisumu livestock owners group recalled milk that had arrived in the center of town, from the Rift Valley region, was poured into the streets after the fresh milk ban was announced. Another livestock owner in Kisumu stated that they began to fear RVFV when a human in Siaya became infected. An SST in Kisumu also recalled this outbreak and added that everyone feared eating meat at that time. In Kisumu East sub-county, at least three livestock owners recalled reactive vaccination of their urban livestock.

A slaughterhouse worker in Kisumu noted that he was never told how to identify an animal that was infected with RVFV over the course of the outbreak, and instead heard about it on the radio. Only one slaughterhouse worker in Ukunda thought he had previously seen signs of RVF in animals and reported that in 2010 there were numerous deaths in cattle with blood-tinged mucus draining from the nose, but this was never confirmed to be RVFV. Those that had not experienced RVF commonly confused it with other fevers and diseases, especially when the other disease has “fever” in the name, for example East Coast fever and yellow fever. Slaughterhouse workers pointed out that when you see something abnormal in the carcass it is challenging to know exactly what disease it is. Other participants didn’t know the disease but attempted to understand the connection with RVFV with the Rift Valley region of Kenya.

“*When the announcement came up about RVF*, *even I can say the milk from Nandi was everywhere here in Kisumu…People just stopped taking the milk*. *The meat stayed because you don’t know where the butchery owner gets their meat from or where they bought*, *but when it comes to milk*, *many people stopped buying*. *Most of the people shifted to the processed milk*.*” – Kisumu LO3 R10*

b. **Insight for future RVFV human vaccination of high risk groups**

When asked about their potential future willingness to be vaccinated against RVFV, there were numerous visual cues observed in every group that suggested a sensitive topic had been raised. Participants initially requested more information about the disease and ingredients in the vaccine before they would accept an injection. Ultimately, the consensus agreement for the majority (greater than 80%) of participants believed that vaccines worked to prevent diseases, and people would be willing to accept a vaccine if they understood their risk. At the slaughterhouse, they were keener to be vaccinated, with some requesting vaccination the same day of the FGD, because they feared high consequence diseases. In Ukunda, livestock owner willingness to be vaccinated was linked to understanding that livestock associated groups were at higher risk than the general community.

“*It’s a must I know what will this vaccine do to me*, *and what is Rift Valley fever*? *After knowing what Rift Valley fever is*, *I might agree*, *but before that I would not accept the vaccine*.*”*

– Kisumu SH3 R7

“*So*, *if you bring that vaccine*, *do you vaccinate everyone in the homestead or just those who graze or look after the animals*? *Because if it can affect me*, *it can also affect my children and the family*. *So how you go about it is the most important thing”*

– *Kisumu LO3 R10*

While there was indeed a high acceptance rate, the main barriers were a lack of a perceived immediate threat, fear of long-term side effects, and confusion with livestock vaccinations. The livestock owners in Ukunda tended to agree that there should be sick animals in their area before they were to accept a vaccination themselves. More hesitancy was present in Ukunda compared to Kisumu as livestock owners were concerned that they could personally receive a cattle injection. A livestock owner in Ukunda said it would be impossible to get people to accept an injection before the disease arrives in their area. In Ukunda, one woman expressed concern that the vaccination could cause infertility and all groups wanted to know the potential negative consequences of this before receiving a vaccine.

“*Without seeing the disease even if you say you are going to pay me to be vaccinated*, *still I won’t accept unless the animals here are sick and can transmit to humans”*

– Ukunda LO1 R1

“*Cows*, *maybe their immunity is higher than that of human beings*, *so the vaccine that would be administered to cow is it the same as the one that would be administered to me*? *Are we going to run in the same line as that*? *The same injection for cows is the same as my injection*?*”*

– *Kisumu LO4 R4*

## Discussion

This qualitative study has highlighted factors relevant to a theoretical understanding of urban RVF ecology and presents key factors and vulnerabilities in urban livestock systems that could support successful introduction of RVFV and other zoonoses via an infected animal and subsequent transmission to susceptible urban hosts. These risks are intertwined with personal sentiments, challenges faced by the urban livestock community, and private business pressure at the slaughterhouse, and these contributing factors warrant inclusion of this perspective in planning future urban risk mitigation. Integrated approaches to surveillance could be expected to allow for earlier and targeted approaches to identifying urban RVFV cases.

RVFV has demonstrated profound adaptability to many different ecological conditions, mammalian hosts, and arthropod vectors [31]. With the possibility of an imported animal seeding an infection and local herds maintaining or amplifying RVFV, urban disease ecology may differ from rural areas and current measures and surveillance approaches do not account for this. For example, RVFV primary vectors in East Africa are thought to be floodwater *Aedes* spp. (primarily *Ae*. *vexans and mcintoshi*), which are not typically associated with urban areas like other *Aedes* spp. (*Ae*. *aegypti and albopictus*) known to transmit dengue and chikungunya viruses. Thus, little research on these other *Aedes spp*. has been caried out in urban and peri-urban areas of endemic RVFV countries where land use change has rapidly shifted water runoff and drainage capacity. In rural areas, floodwater *Aedes* spp., are thought to play a significant role in outbreak initiation, but perhaps, outbreak initiation dynamics differs in the urban environment. For example, urban outbreaks could be initiated by introduction of a viremic animal at the slaughterhouse and viral amplification could occur in the holding pen before eventual spillover to the human population or local urban livestock. The force of infection for this process may be increased compared to rural area given the host density and could be driven by any of the known dominant mosquito vectors that have varied across outbreaks [22,40]. The potential for an undetected introduction coupled with RVF’s complex epidemiology requires that prevention measures account for all aspects of transmission including human behaviors that affect risk, host interactions with vectors, and opportunities for urban resident animals to be exposed.

With insight from previous RVFV disease introductions and other urban arbovirus outbreaks [8,10,14,25], this qualitative study foreshadows how some key high risk populations in urban centers may identify and respond to an urban introduced RVFV viremic animal. For recognizing the likely first urban livestock cases, slaughterhouses are a key entry point for RVFV as animals enter in high volume from a wide geographical range. Passive syndromic surveillance of livestock alone would be expected to fail in this system as the herd context is required for detecting the most recognizable signs of clinical RVF, abortion storms and death of young animals. Most animals entering the urban center are adult cattle, which often have inapparent infections [41] and could inadvertently infect slaughterhouse workers, local livestock, or urban mosquitos near the slaughterhouse. The risk to local urban livestock exposure from these imported animals is highlighted in theme one-b (Nutritional demands drive urban influx of livestock and animal products) as imported animals have ample opportunity for mixing, either directly or within vector flight range, with non-local urban resident animals. If local animals were to become infected, the congested living conditions of humans and livestock in the urban setting may lower the spillover threshold to humans. These situational grazers arrive when there is drought at their home and they aim to move towards areas with heavy rainfall over time, so could be exposed while in route before arriving near to our urban sites. This is a key time for when where urban residing livestock may encounter high risk animals and the frequency and patterns of these movements may change with the pressure of climatic change. Notably, urban livestock roaming is primarily due to the lack of feed availability, and in the case of a regional outbreak, supporting nutritional demands of urban animals could limit unauthorized (or locally authorized) movement. Moreover, if RVFV is already present in urban vectors, livestock movement bans may be too delayed to have significant effects on the outbreak magnitude and the subsequent threat to livelihoods may not be worthwhile. Thus, testing imported animals for RVFV and tracing their origins would be the most effective way to identify an urban introduction and would contribute to a better understanding of the outbreak’s spatial boundaries.

Important when considering direct urban community transmission of RVFV independent of livestock ownership, theme two (*Consumption of fresh animal products by the urban community*) demonstrates retention of cultural practices at the two urban sites that could lead to RVFV transmission: consumption of raw blood and fresh milk. Pooled blood purchased directly from slaughterhouses poses a risk to everyone that handles and prepares the product as RVFV is well documented to aerosolize from infected blood, and this exposure mechanism has been linked to more severe infections [42,43]. The exchange of blood from the slaughterhouse also shifts risk into the community where risk factors may be less recognizable, and further complicates differentiating human RVF disease clinically at health centers from other febrile diseases, including malaria and meningitis [44]. As vector exposure is a near ubiquitous risk factor, it is often challenging to attribute exposure to vectors alone in retrospective recall questionnaires. Slaughtering, and consuming sick animals has been associated with an increased risk of death, yet severe infections have occurred in humans without any known animal contact [45]. When we explored if our participants had personal experience with RVFV outbreaks specifically, their responses suggested that public health messaging from previous RVFV outbreaks at our study sites had been primarily focused on consumption of meat and milk, rather than vector exposure, and this is how participants evaluated their risk. This is in contrast to a study conducted in pastoralist communities of Northeastern Kenya where the majority of participants believed RVFV infection to be from mosquito bites rather than from consumption of milk, meat, and blood [46]. The authors of that qualitative study reported that while their participants had heard of consumption related risks, experiences didn’t match their health outcomes and, to them, consumption of meat or milk was not risky for contracting RVFV. In the urban setting, meat and milk consumption is common and there are multiple actors along value chains, for example, producers, middle businesspeople, processors, and inspectors, before ultimately the consumers, which can lead to miscommunication about origin and safety of these products. There may also be implications for blood waste management that contribute to urban transmission dynamics due to the high volume of slaughtered animals and limited physical space because inadequately managed wastewater can breed mosquitos, but we did not investigate this in our study [47]. Data on viral presence and stability in blood after slaughter, milk and dairy products, and wastewater from urban slaughterhouses could help quantify these public health risks and allow for more specific and directed policy that could be used to build trust and/or be leveraged for surveillance.

We found little differences between the two study sites which may suggest similar urban challenges in livestock systems. However, this study has several additional limitations and results may not be able to be extrapolated to other urban areas in Kenya. We attempted to represent the urban livestock owners well, but our participant selection was not randomized. By purposefully selecting dairy owners from one sub-county of Kisumu, we may have introduced selection bias in our understanding of fresh milk consumption in the greater urban area. Another major limitation in this study was only including the veterinarian meat inspector at the selected slaughterhouse and we may have excluded differing viewpoints from other public health veterinarians. All in all, this was an exploratory study, and the objectives cannot be answered by qualitative methodologies alone. Nonetheless, this initial study provides a theoretical framework that could be expanded on in other locations to better understand similarities between urban areas RVFV risk to guide future targeted field epidemiology studies in urban centers.

Our study demonstrated regulatory vulnerabilities in theme three (*High reliance on veterinarian to determine safety and vulnerable regulations*) including selling of unofficially slaughtered meat and unauthorized animal movements that should be considered in urban outbreak investigations and be integrated into future preventive measures. People’s perception of risk impacts their behaviors, and this can either increase the chance of exposure (drinking blood) or be protective (refusing to slaughter dead animals). Therefore, understanding perceptions and challenges experienced by high risk groups allows for a more realistic understanding of why regulatory vulnerabilities persist. We highlighted that these groups had moderate knowledge of zoonoses and we determined that current slaughterhouse regulations are focused on common bacterial and parasitic diseases relevant to food safety. This was demonstrated by the participants mentioning hygiene and visual inspection of organs as key measures to determine food safety and they affirmed that if animals are inspected, they must be safe. Reliance on the postmortem examination leads slaughterhouse workers to believe that they must slaughter the animal to determine its ailment, which could result in their own personal exposure to zoonoses during the slaughtering process. With few individual exceptions, group consensus determined risk to be lower in urban areas compared to rural areas because of improved urban hygiene. Still, fear filled the gaps in participant knowledge, as they requested to learn more about their risk of RVFV.

Awareness building and education of high risk groups could contribute to improved future cooperation with disease countermeasures. Messaging would be best focused on the key activities associated with urban slaughterhouse risk and risks in the greater community including handling and consumption of fresh milk and blood, accidental consumption of meat from a sick animal, and urban vector exposure. Awareness for diseases with unconventional transmission mechanisms, such as direct aerosolization from animal blood to humans, should be accomplished in parallel with continued management of other prevalent bacterial and parasitic diseases. In understanding of existing zoonotic disease countermeasures, we explored high risk groups willingness to personally be vaccinated for RVFV if one were to become available for use in humans. Groups requested specific information about the vaccine’s safety and efficacy, and the point raised by one of the livestock owners regarding their concern for receiving a cattle vaccine will be an important point to emphasize in future sensitization efforts for One Health vaccine campaigns.

Formalizing meat trade in Kenya by requiring a stamp of inspection from qualified veterinarians has undoubtedly improved detection of key zoonotic diseases and contributed to the improvement of Kenya’s meat safety, although in agreement with other studies, conditions do not always align with Kenya’s Meat Control Act [48]. All meat inspector veterinarians in this study shared personal stories about the importance of oversight when condemning a carcass, yet livestock owners reported that corruption and falsification in meat certification persists. Community-based education of vulnerable groups may alter personal decisions to partake in this risky behavior. For urban introduction of RVFV, slaughterhouse workers and their business partners may be the first line of defense in outbreak control. Full reliance on veterinarians to make key decisions for slaughtering and consumption undermines the ability for slaughterhouse workers to be leveraged in reporting sick animals. In our IDIs, veterinarians understood this public health responsibility, but added that there was insufficient diagnostic support for RVFV, and they feared missing the diagnosis. Underequipping and underpreparing urban meat inspector veterinarians for introduction of RVF is perhaps the greatest missed opportunity to protect urban public health. Furthermore, local One Health initiatives and better administrative cooperation between human health providers and veterinarians are required even before any acute cases are detected locally. If RVFV were to enter an urban center of an endemic region and establish an urban transmission cycle, the human health, and economic effects from loss of livestock would be devastating and concern for re-emergence after would persist every subsequent flooding event. Urban livestock markets would be significantly disrupted and pressure to bypass regulations to maintain livelihoods would be high. Greater understanding of these potential risks is warranted now, before the first urban RVF outbreak.

### Conclusion: Implications for future policy to prevent and control urban RVF

This study has informed understanding of potential pathways of introduction and transmission in the context of current practices and urban high risk groups’ perceived risk. Our data suggest that urban slaughterhouses may be a key entry point for RVFV, and current systems may not have the capacity for detection given the lack of apparent clinical signs and need for access to diagnostic assays. At the slaughterhouse and in the community, there is a high degree of reliance on veterinarians to determine safety; however, veterinarians report difficulty accessing these important confirmatory diagnostic tools. Meanwhile, with the focus on hygiene and visual post-mortem inspection, RVFV may be inadvertently transmitted to a slaughterhouse worker and the dense host-vector environment in urban centers may provide a means for infection of local urban resident animals that live near to households.

Finally, we have demonstrated that high risk populations have experienced other zoonotic disease countermeasures which may foreshadow their reactions to future RVF urban control measures. Tracing the pathway of blood, organs, and fresh milk could inform risk in the greater community and inform development of inclusive surveillance programs including diagnostic support at human health centers in areas with detected livestock cases. Additional measures to prepare urban areas for RVF include education of high risk groups, community-based awareness building, preventive livestock vaccination, scenario planning, and surveillance. Passive surveillance is expected to be less reliable in urban areas without the context of large herds in rural areas, and therefore, we recommend active surveillance, particularly during high risk times, and development of laboratory procedures for pooled samples to expand cost-effectiveness. To increase the likelihood of a feasible surveillance system, such active surveillance should ideally be integrated into activates already existing in animal systems, such as gathering for slaughter or at marketplaces. The slaughterhouse soup traders group confirmed that private blood sales from the slaughterhouse to consumers occurs and these products could be intercepted and screened for RVFV. Inherently, any integrated surveillance system will be highly specific to the area, and participation from local stakeholders in any given urban area will be the ultimate key to success and sustainability.

Given the profound potential downstream effects from introduction of a viremic animal to the urban center, an early and rapid response will be required to give urban centers the best opportunity at avoiding infection of urban hosts and vectors and possible initiation of an urban transmission cycle. It is not currently known if an urban transmission cycle is sustainable, and quantifying mosquito vector abundance and diversity at urban slaughterhouses could guide understanding of this process and advise localized vector control strategies. In the meantime, larval source reduction remains important in arboviral disease control, especially in the urban setting (49). Integrating high risk groups’ perspective for zoonotic diseases into planning of future preventive measures in urban areas would be expected to be more efficacious than top down approaches and would lower the risk of marginalizing vulnerable livestock associated groups during an outbreak. Additionally, a digital record keeping system of urban animal imports could allow veterinarians to carry out local surveillance on suspect animals that enter from high risk markets known to source animals within high risk zones. Affiliates of the slaughterhouse, veterinarians, and livestock owners would likely be on the frontlines to be able to identify critical early introduced livestock cases in the urban setting and, therefore, empowering these groups to monitor animals originating from markets with higher risk and could assist urban slaughterhouse veterinarians in Kenya that already report overwhelming, fast-paced workloads. However, as previously mentioned, most animals in the slaughterhouse setting will not show clinical signs and thus, expanding access to diagnostics remain a top priority for quantifying risk. Urban areas must prepare surveillance frameworks and contingency plans for managing RVFV outbreaks and adapt the context of health messaging from other areas to the perceptions of their own communities.
